# Anti-Aging Effects of Quercetin in Cladocera *Simocephalus vetulus* Using Proteomics

**DOI:** 10.1021/acsomega.2c08242

**Published:** 2023-05-09

**Authors:** Ying Yang, Yiming Li, Xinglin Du, Zhiquan Liu, Chenxi Zhu, Weiping Mao, Guoxing Liu, Qichen Jiang

**Affiliations:** † Freshwater Fisheries Research Institute of Jiangsu Province, 79 Chating East Street, Nanjing 210017, China; ‡ Institute of Biochemistry and Biological Products, School of Life Sciences, Nanjing Normal University, Nanjing 210046, China; § The Low Temperature Germplasm Bank of Important Economic Fish of Jiangsu Provincial Science and Technology Resources (Agricultural Germplasm Resources) Coordination Service Platform, Freshwater Fisheries Research Institute of Jiangsu Province, Nanjing 210017, China; ∥ Fishery Machinery and Instrument Research Institute, Chinese Academy of Fisheries Sciences, Shanghai 200092, China; ⊥ School of Life Sciences, East China Normal University, Shanghai 200241, China; # School of Life and Environmental Sciences, Hangzhou Normal University, Hangzhou 311121, Zhejiang, China; ¶ School of Engineering, Hangzhou Normal University, Hangzhou 310018, Zhejiang, China

## Abstract

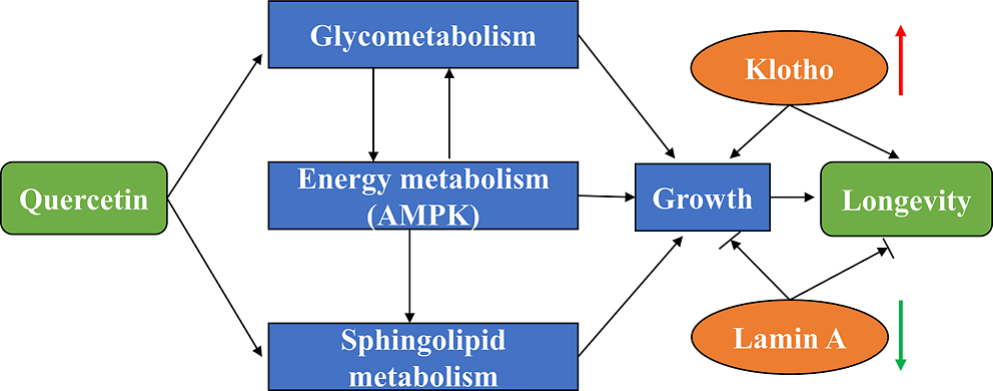

Quercetin is a flavonoid widely found in food and traditional herbs. In this study, we evaluated the anti-aging effects of quercetin on *Simocephalus vetulus* (*S. vetulus*) by assessing lifespan and growth parameters and analyzed the differentially expressed proteins and crucial pathways associated with quercetin activity using proteomics. The results demonstrated that, at a concentration of 1 mg/L, quercetin significantly prolonged the average and maximal lifespans of *S. vetulus* and increased the net reproduction rate slightly. The proteomics-based analysis revealed 156 differently expressed proteins, with 84 being significantly upregulated and 72 significantly downregulated. The protein functions were identified as being associated with glycometabolism, energy metabolism, and sphingolipid metabolism pathways, and the key enzyme activity and related gene expression, such that of *AMPK*, supported the importance of these pathways in the anti-aging activity of quercetin. In addition, quercetin was found to regulate the anti-aging-related proteins Lamin A and Klotho directly. Our results increased the understanding of quercetin’s anti-aging effects.

## 1 Introduction

Flavonoids are secondary metabolites that widely exist in plants and have attracted attention for their diverse biological and pharmacological activities.^1,2^ Quercetin is one of the most common dietary flavonoids present in vegetables, fruits, and Chinese herbal medicines.^3−5^ Quercetin has a variety of physiological effects, including anti-cancer, antibacterial, and anti-inflammatory activities, and offers cardiovascular protection. It is widely known for its strong antioxidant properties.^6^ Recently, quercetin has been found to prolong the lifespan of various organisms such as yeast, *Caenorhabditis elegans* (*C. elegans*), *Drosophila*, and mice^7−11^ and to directly increase the expression of longevity genes such as *Sir*1 in human aging skin fibroblasts.^12,13^ However, to the best of our knowledge, the anti-aging effects of quercetin in aquatic animals are largely unknown, and the mechanism of its anti-aging activity remains to be explored.

Cladocerans, widely distributed aquatic zooplankton, have numerous characteristics that make them a novel and versatile model for aging research. For instance, their small size makes them easy to cultivate, and they have short lifespans. The parthenogenetic mode of reproduction in cladocerans allows them to produce a large number of genetically identical individuals.^14^ Furthermore, they harbor significantly more genes than *Drosophila* and *C. elegans*, raising the possibility that additional genes with relevance to human anti-aging could be identified in them.^15^*Simocephalus vetulus* (*S. vetulus*) is a very widely distributed cladoceran and is relatively large and easy to handle compared to many other cladocerans.^16^

Proteomics technology is an effective tool for obtaining convincing experimental data. It can be used to identify the differentially expressed proteins (DEPs) in individuals under drug treatment and reveal the most important molecular pathways affected.^17^ Although there have been some reports about the anti-aging regulatory mechanism of quercetin in organisms,^18,19^ profile information on proteins regulated by quercetin with respect to anti-aging activity remains lacking, especially in aquatic organisms.

The aim of this study was to evaluate the effects of quercetin on the growth and lifespan of *S. vetulus*, explore the anti-aging effects of quercetin in cladocerans, and investigate the molecular mechanism using proteomics. We analyzed the average and maximum lifespans and growth parameters of *S. vetulus*, identified and quantified the DEPs in quercetin-treated and control groups, and screened key proteins and pathways. In addition, we assessed the enzyme activities and gene expression after 7, 14, and 21 d of quercetin treatment in *S. vetulus*, respectively. The present study provided new information about the anti-aging mechanism of quercetin.

## 2 Materials and Methods

### 2.1 Medicines and Reagents

Quercetin (purity >98%) was purchased from Sigma-Aldrich (Shanghai, China) and stored at −20 °C away from light. Quercetin was dissolved in dimethyl sulfoxide (Jiancheng, Nanjing, China) before use.

### 2.2 Animal Culture

*S. vetulus* individuals were cultured and maintained at 25 ± 1 °C in tap water with continuous aeration for 24 h before use, with a 12 h light/12 h dark photoperiod, and fed green algae, *Chlorella pyrenoidosa*. *S. vetulus* used in our experiment were originally collected from the Grand Canal (Wuxi, China) and have been continuously cultivated in our laboratory for more than 2 years. The healthy neonates (<24 h) from their third brood were collected and used in these experiments.

### 2.3 Lifespan Statistics and Growth Parameter Tests of *S. vetulus*

Individuals were kept in 500 mL beakers during the experiment, and according to a preliminary experiment, five groups subjected to different concentrations (0, 1, 2.5, 5, and 10 mg/L) of quercetin were set. There were three replicates of each concentration group, and each replicate contained 30 *S. vetulus* juveniles (<24 h). The new neonates were removed, and the dead individuals were discarded when the solution was changed each day. The number of the deaths was recorded. The experiment continued until all individuals died; then, survival rates were calculated, and survival curves were drawn. In addition, the number of surviving individuals and the number and time of births were recorded during the experiment. We then calculated the net reproduction rate, intrinsic growth rate, and generation time.^20^

### 2.4 Proteomics Analysis

#### 2.4.1 Sample Collection and Protein Extraction

After 7 d, the group exposed to 1 mg/L quercetin and the control group were collected for proteomics analysis. There were three replicate groups for each treatment. A sample (0.1 g) of *S. vetulus* individuals was collected from each replicate and transferred quickly into liquid nitrogen for protein extraction. Frozen samples were ground to powder and transferred to 1.5 mL centrifuge tubes. Phenolic extractant and the protease inhibitor phenylmethanesulfonyl fluoride were then added to the tubes. After sonication, an equal volume of phenol-Tris-HCl was added, and the saturated solution was well mixed at 7100 rpm, centrifuged at 4 °C for 10 min, and the supernatant was collected. After sonication, ammonium acetate-methanol and acetone were added to the supernatant, and the precipitates were collected and dried. The total protein solution was obtained by dissolving the dried sample, and the concentration of protein was detected using bicinchoninic acid.^21^

#### 2.4.2 Protein Digestion and TMT Labeling

Reducing agent buffer (120 μL) was added to the ultrafiltration tube containing 100 μg of the protein sample and left to react at 60 °C for 1 h. IAA was then added to the reaction solution to give a final concentration of 50 mM, and the tube was kept away from light for 40 min before being centrifuged at 12,000 rpm and 4 °C for 20 min. Next, triethylamonium bicarbonate (TEAB) buffer and trypsin solution were added, and the tube was centrifuged under the same conditions to collect the peptides. Subsequently, more TEAB buffer was added to the ultrafiltration tube before centrifugation, and finally, the solution was lyophilized. TEAB buffer was then added to the lyophilized samples and mixed well before TMT labeling reactions were performed. TMT reagent and anhydrous acetonitrile were added to the prepared samples. After 1 h, 5% hydroxylamine was added to stop the reaction for 15 min, and the samples were stored at −80 °C after lyophilization.

#### 2.4.3 Reversed-Phase Liquid Chromatography

An Agilent 1100 HPLC system was used for reversed-phase liquid chromatography (RPLC) separation with an Agilent Zorbax Extend-C18 column (5 μm, 150 nm × 2.1 mm) and UV detection at 210 nm and 280 nm. Mobile phase A and mobile phase B were set to ACN-H_2_O (2:98, v/v) and ACN-H_2_O (90:10, v/v), respectively, and the flow rate was set to 300 μL/min. The gradient elution conditions were as follows: 0–8 min, 98% A; 8.00–8.01 min, 98–95% A; 8.01–38 min, 95–75% A; 38–50 min, 75–60% A; 50–50.01 min, 60–10% A; 50.01–60 min, 10% A; 60–60.01 min, 10–98% A; and 60.01–65 min, 98% A. The samples were collected between 8 and 50 min; the eluate buffer was collected every minute into centrifuge tubes numbered 1–15 and cycled in this order until the end of the gradient. After collection, frozen samples were prepared for mass spectrometry.

#### 2.4.4 Mass Spectrometry

Samples were loaded and separated on a C18 column (15 cm × 75 μm) using an EASY-nLC 1200 system (Thermo Fisher Scientific, USA). The gradient elution conditions were as follows: 0–40 min, 5–30% B; 40–54 min, 30–50% B; 54–55 min, 50–100% B; and 55–60 min, 100% B. Mobile phase A and mobile phase B were H_2_O-FA (99.9; 0.1, v/v) and ACN-H_2_O-FA (80; 19.9 0.1, v/v/v), respectively. The mass resolution was set to 70,000, and the automatic gain control value was set to 1 × 10^6^. The system was set to scan the full mass range at 300–1600 *m*/*z*, and the 10 highest peaks were identified with MS/MS. All MS/MS spectra were collected using high-energy collisional fragmentation, with the collision energy set to 32. MS/MS resolution, automatic gain control, maximum ion accumulation time, and dynamic exclusion time were set to 175,000, 2 × 10^5^, 80 ms, and 30 s, respectively.

#### 2.4.5 Protein Quantification and Bioinformatics Analysis

The data were analyzed using Proteome Discoverer 2.2 software (Thermo Fisher Scientific, USA). The UniProt-daphniidae database was used to search for proteins, and the false positive rate of peptide identification was controlled below 1%. The specific parameters were set to: trypsinization digestion specificity for the database search, alkylation of cysteine for fixed modifications, and TMT6-plex for protein quantification. In addition, missed cleavages were set to 2, MS 1 tolerance was set to 20 ppm, and MS 2 tolerance was set to 10 ppm. The date of access of the database is June 2019, and the number of entries is 1,10,177.

According to the sequest HT score >0, unique peptides ≥1, and criteria for removing blank values, credible proteins were screened from the raw data. Each group of data was screened for incredible proteins to obtain the fold-change (FC) value and *p*-value of the comparison group. The standards of FC > 1.2 or FC < 5/6 and *p*-value <0.05 were used to screen the DEPs. R (package 4.2.0) was used for data statistical analysis, and ggplot2 (3.3.0) was used for image visualization. The normalization method was the algorithm that comes with software Proteome Discoverer 2.2. The Benjamini and Hochberg algorithm was used to adjust the *p*-value. The subsequent biological function analysis was significantly based on the DEPs. To analyze the function of the DEPs, the Omics Bean omic data integrated analysis cloud platform was used to determine Gene Ontology (GO) functional annotation and enrichment and Kyoto Encyclopedia of Genes and Genomes (KEGG) enrichment. The method of enrichment analysis used the species protein as the background list and screened a differential protein list as the candidate list. The hypergeometric distribution test was used to calculate the *p*-value, which represents the significance of functional enrichment in the differential protein list. The *P*-value was corrected for false discovery rate using the Benjamini and Hochberg multiple testing correction. The GO functional annotation included three categories of analysis: biological process, cellular component, and molecular function.

### 2.5 Enzyme Activity Detection

After quercetin treatment, *S. vetulus* individuals cultured at 0, 1, 2.5, 5, and 10 mg/L quercetin concentrations for 7 d and 14 d, and individuals cultured at 0 and 1 mg/L quercetin concentrations for 21 d were placed in 2 mL centrifuge tubes and homogenized with physiological saline. An electric homogenizer was used to prepare the homogenate on ice, and the supernatant was obtained by centrifuging at 2000 rpm/min for 15 min at 4 °C and was used for the enzyme test. The activity of amylase was calculated from the optical density (OD) of the colored complexes formed by the combination of iodine and starch.^22^ The activity of cellulase was determined by the color reaction of the reducing sugar produced by cellulolysis and dinitrosalicylic acid.^23^

### 2.6 RNA Isolation and Quantitative RT-PCR

At the end of the quercetin treatments, at 7 d (0, 1, 2.5, 5, and 10 mg/L quercetin concentrations), 14 d (0, 1, 2.5, 5, and 10 mg/L quercetin concentrations), and 21 d (0 and 1 mg/L quercetin concentrations), whole *S. vetulus* individuals were homogenized on ice, and the total RNA was extracted using the TRIzol reagent (TransGen, Beijing, China). The purity and integrity of the RNA were detected by 1% agarose gel electrophoresis and with a NanoDrop 2000 system (Thermo Fisher Scientific, Wilmington, DE, USA), respectively. RNAs with A260/A280 ratios (ratios of absorbance at 260 nm to that at 280 nm) in the range of 1.8–2.2 were used to synthesize cDNA. The cDNA was reverse-transcribed with a Prime-Script RT reagent kit (Takara, Japan) following a standardized procedure. The reverse transcription program was performed at 37 °C for 15 min and 85 °C for 5 s to synthesize the first-strand cDNA.

**Figure 1:**
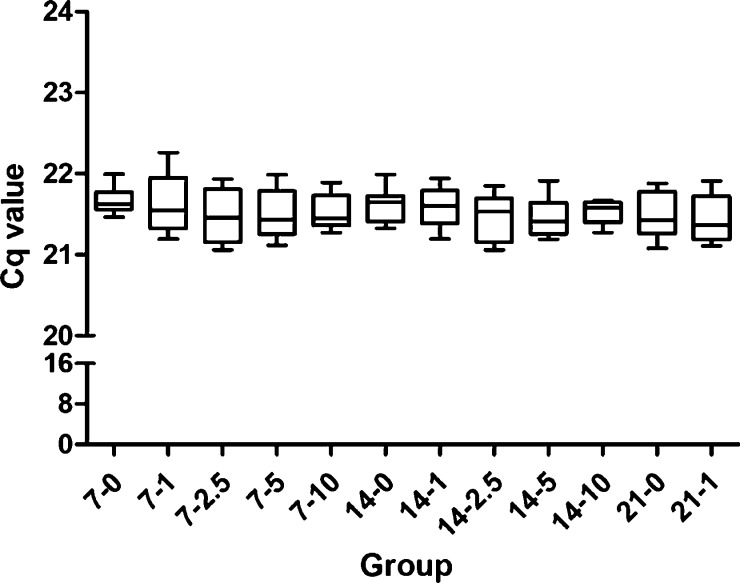
No significant difference between reference gene stability data.

Quantitative RT-PCR was performed using Eppendorf Mastercycler ep realplex RT-PCR (Eppendorf, Germany). A fluorescent quantification dye was used in this study, and the specific product used was a qPCR super mix (Takara, Japan). All operations were carried out according to the manufacturer’s instructions. The PCR solution included 10 μL of SYBR, 7.2 μL of ddH_2_O, and 1.2 μL of the cDNA, with 0. 8 μL of each 10 μM primer. *AMP*-activated protein kinase genes (*AMPK*α, *AMPK*β, and *AMPK*γ) were selected for detection based on the effects observed during the proteomics experiment. Primers were designed using Primer Premier 5 software. The 18*s* gene was used as the internal reference. All RT-qPCR experiments were performed in triplicate and normalized to the control gene. The primer sequences used for the gene expression experiments are listed in Table 1. The stability of 18*s* as the internal reference under different quercetin conditions is shown in Figure 1. Gene expression levels were calculated using the 2^–ΔΔCt^ method.^24^

**Table 1:** Primer Sequences Used in the qPCR

gene	primer sequence (5′-3′)	GC content (%)	annealing temperature (°C)	expected amplicon size
18*s-F*	TGCCAGCCGCTTTAGGAGTA	55	58	94 bp
18*s-R*	CCCGAACGCAGTTGTTTTGT	50	60	
*AMPK*α*-F*	GACTTTGAGTGGAAGACGGTAA	45	55	198 bp
*AMPK*α*-R*	TGAAAACTGTGATTGCGACA	40	52	
*AMPK*β*-F*	GGGCGATGATGACGACTTTT	50	57	232 bp
*AMPK*β*-R*	ATGGCTCTTCACCATTGGTATG	45	57	
*AMPK*γ*-F*	TTGAGGCAAGCCAATGAACA	45	57	128 bp
*AMPK*γ*-R*	TCCACAACAACCAAACGATG	45	55	

### 2.7 Data Analysis

All data are presented as mean ± standard deviation (SD). To determine the differences between the control and quercetin groups, one-way analysis of variance (ANOVA) and Tukey’s test were used. The synergistic effect between quercetin concentrations and treatment time was analyzed by two-way ANOVA. Data were analyzed using Origin Pro 9.1 (Origin Lab, Northampton, MA, USA), and graphs were created using Graph Pad 5.0 (Graph Pad Software, La Jolla, CA, USA). *P* < 0.05 was used to indicate the significance of the data.

## 3 Results

### 3.1 Effects of Quercetin on the Lifespan in *S. vetulus*

**Figure 2:**
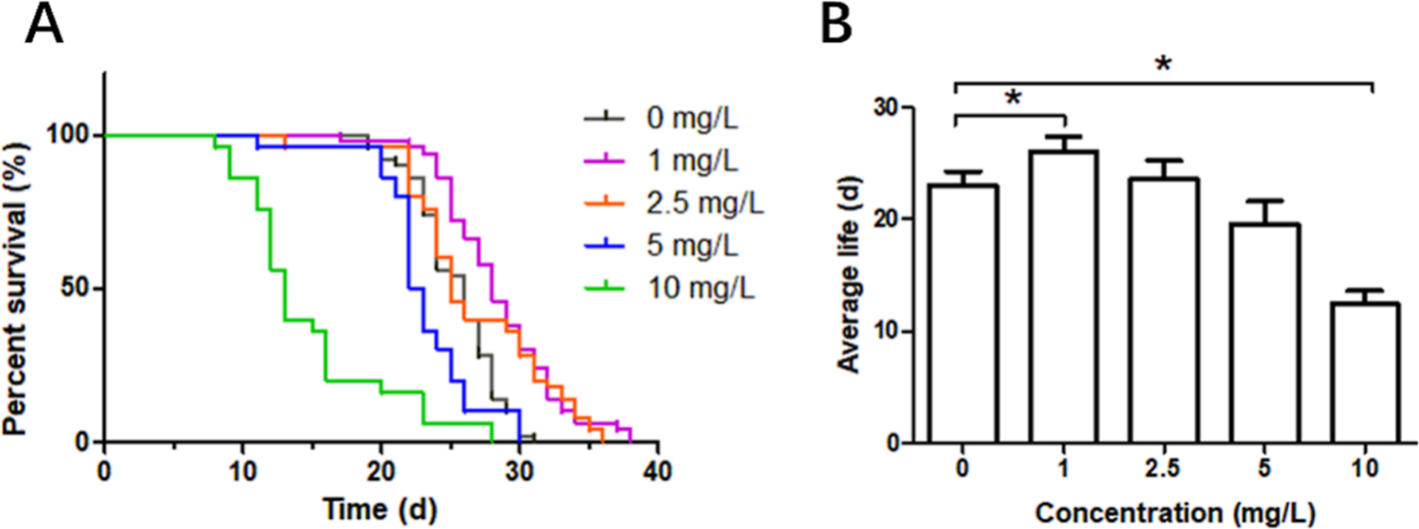
Effects of quercetin on percent survival (%) (A) and average lifespan (B) of *S. vetulus*. Significant differences in the average lifespan compared with the control groups are indicated by asterisks (*P < 0.05).

Under treatment with quercetin at concentrations of 1 and 2.5 mg/L, the maximum lifespan of *S. vetulus* increased significantly. In particular, 1 mg/L quercetin had a very significant effect on the maximum lifespan, which also significantly prolonged the average lifespan (*P* < 0.05). At 10 mg/L quercetin, the maximum lifespan declined, and the average lifespan decreased significantly (*P* < 0.05) (Figure 2).

### 3.2 Effects of Quercetin on Growth Parameters in *S. vetulus*

**Figure 3:**
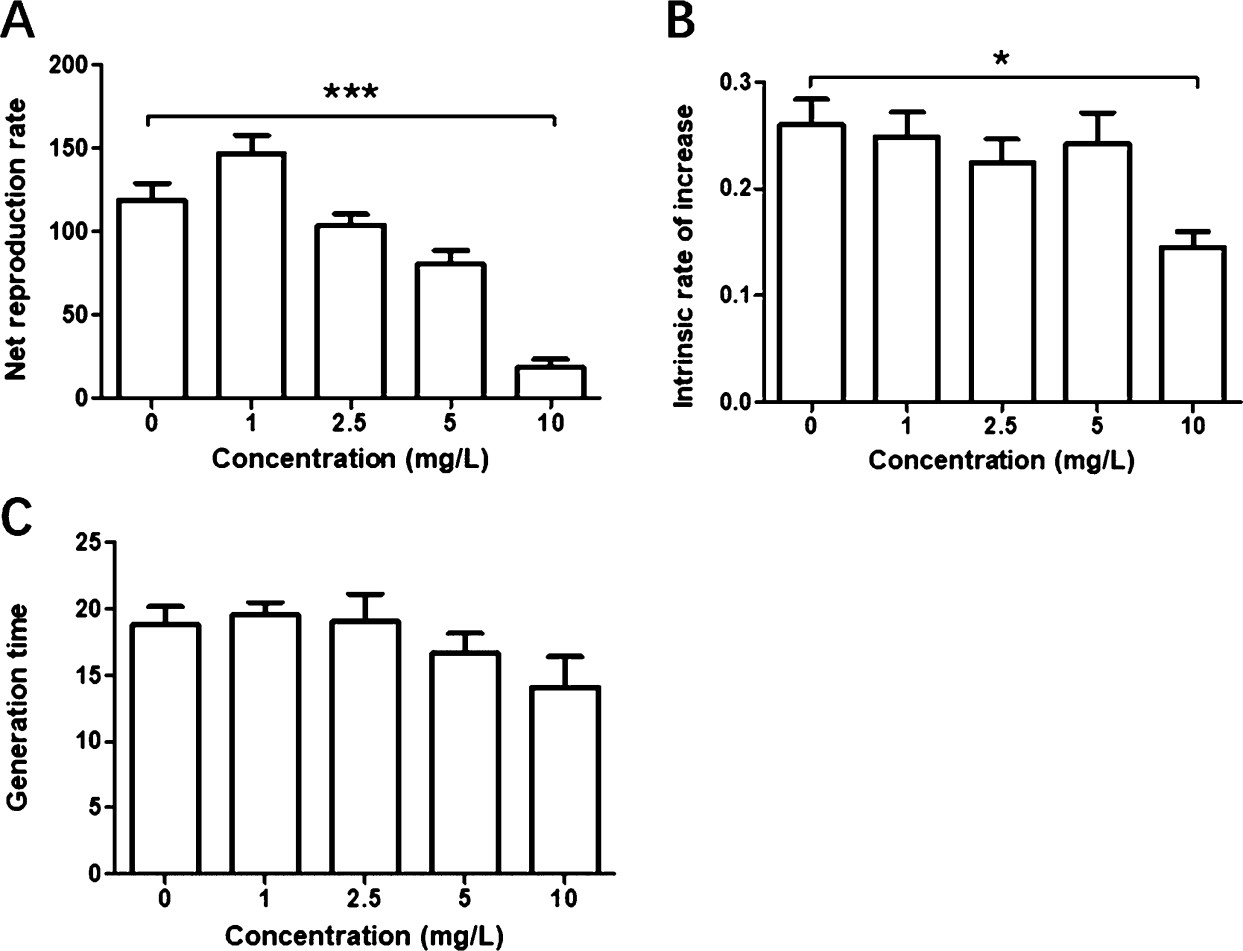
Effects of quercetin on the growth parameters of *S. vetulus*. (A) Net reproduction rate, (B) intrinsic rate of increase, and (C) generation time. Values are presented as mean ± standard deviation (SD). Significant differences from the control groups are indicated by asterisks (*P < 0.05, ^**^P < 0.01, and ^***^P < 0.001).

As shown in Figure 3, the net rate of *S. vetulus* reproduction showed an increasing trend in the 1 mg/L quercetin group, although it was not significant, while it significantly decreased in the 10 mg/L quercetin group compared to that in the control group (*P* < 0.05) (Figure 3A). Concentrations of 1, 2.5, and 5 mg/L quercetin had no significant effect on the intrinsic growth rate and generation time in *S. vetulus* (*P* > 0.05), but the high concentration (10 mg/L) significantly decreased the intrinsic growth rate compared to that in the controls (*P* < 0.05) (Figure 3B,C).

### 3.3 Identification of DEPs in *S. vetulus*

**Figure 4:**
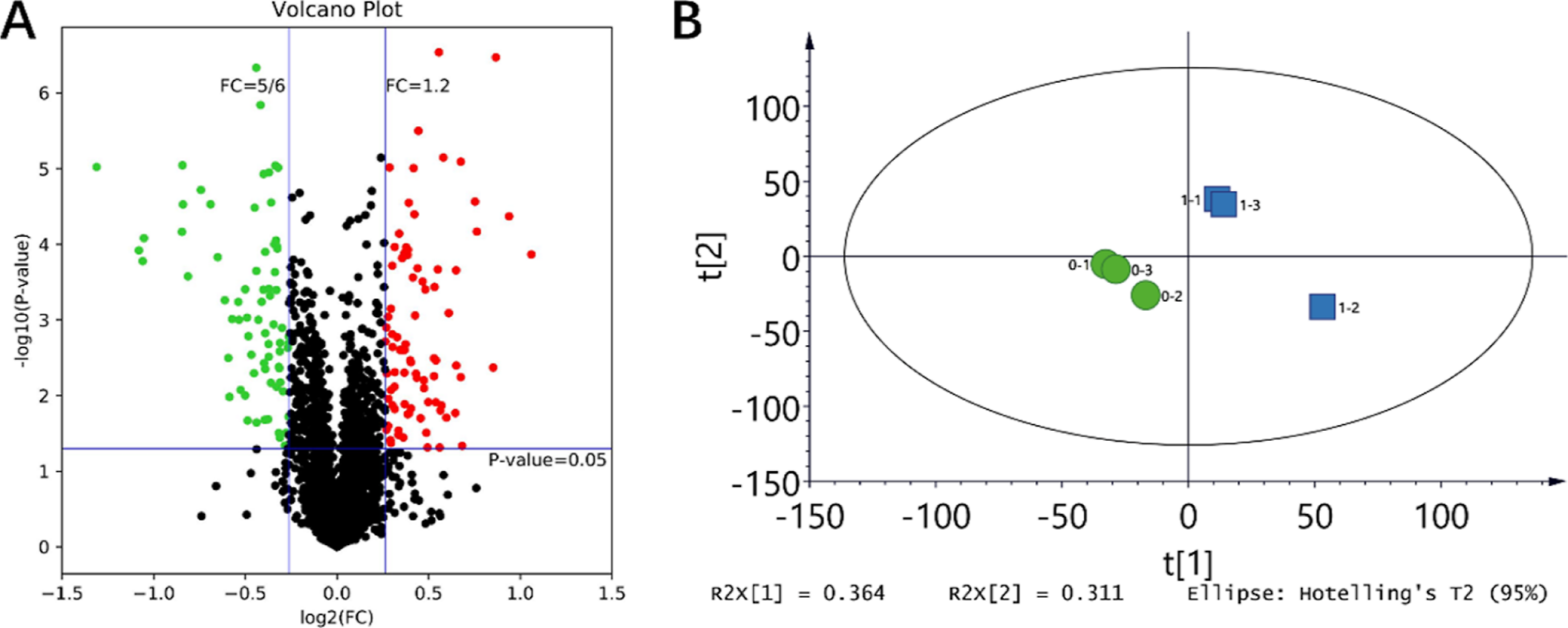
(A) Volcano plot map of DEPs in *S. vetulus* after quercetin treatment, red: upregulated DEPs, green: downregulated DEPs, and black: no significant DEPs. (B) Principal component analysis of DEPs in *S. vetulus* after quercetin treatment.

In total, 2932 credible proteins and 156 significantly DEPs were screened, of which 84 were significantly increased and 72 significantly decreased (Figure 4A). The principal component analysis showed two distinct clusters, with 67.5% of the data variability being explained by the two axes (Figure 4B).

### 3.4 Functional Enrichment Analysis of DEPs in *S. vetulus*

**Figure 5:**
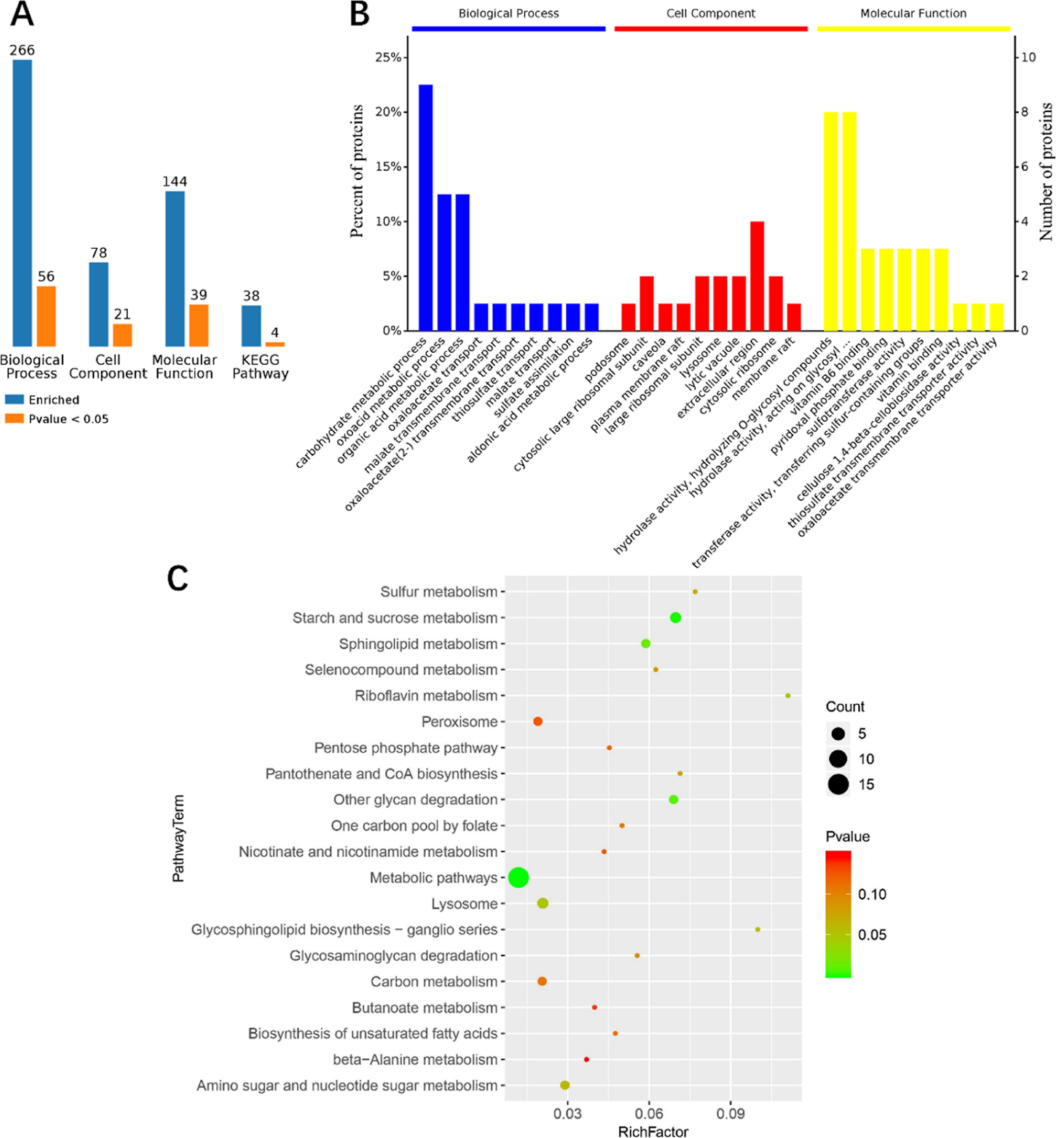
Functional enrichment analysis of DEPs after quercetin treatment. (A) Summary of functional enrichment pathways. (B) GO enrichment: blue, red, and yellow indicate biological progress, cell component, and molecular function, respectively. In each category of entries, the closer to the left, the more significant the difference. (C) KEGG pathway enrichment. The color and size of the solid circles represent the *P* value and the number of proteins enriched in the pathway, respectively.

GO functional analysis showed that 156 DEPs, corresponding to the number of biological process, cellular component, and molecular function items, were 266, 78, and 144, respectively. Among these GO terms, 56 biological processes, 21 cellular components, and 39 molecular functions were significantly enriched (*P* < 0.05) (Figure 5A). In the biological process, the top three categories of DEPs affected were the carbohydrate metabolic process (GO: 0005975), oxoacid metabolic process (GO: 0043436), and organic acid metabolic process (GO: 0006082); the cellular components included podosome (GO: 0002102), cytoplasmic ribosomal component (GO: 0022625), and caveolae (GO: 0005901); and the molecular functions were hydrolase activities, including hydrolyzing O-glycosyl compounds (GO: 0004553) and acting on glycosyl bonds (GO: 0016798) and vitamin B6 binding (GO: 0070279) (Figure 5B).

KEGG pathway enrichment results showed that the DEPs were enriched in 38 pathways, of which four were significantly enriched (*P* < 0.05) (Figure 5A), including energy metabolism pathways, starch and sucrose metabolism, other glycan degradation, and sphingolipid metabolism (Figure 5C).

### 3.5 Amylase and Cellulase Activity and mRNA Expression of *AMPK*

**Figure 6:**
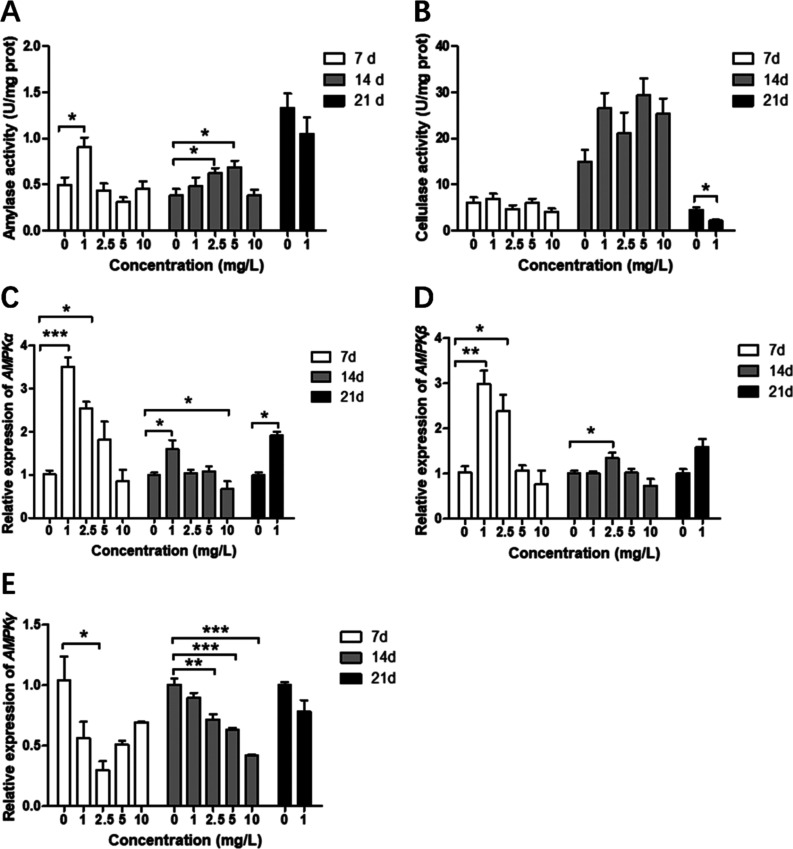
Enzyme activity of (A) amylase, (B) cellulase, and relative gene expression of (C) *AMPK*α, (D) *AMPK*β, and (E) *AMPK*γ in *S. vetulus* after 7, 14, and 21 d of quercetin treatment. Values are presented as mean ± standard deviation (SD) (*n* = 3). Significant differences from the control groups are indicated by asterisks (*P < 0.05, ^**^P < 0.01, and ^***^P < 0.001).

As shown in Figure 6A, compared to the control group, the activity of amylase was significantly increased in the groups treated with low concentrations of quercetin for 7 and 14 d (*P* < 0.05). Cellulase activity showed an increasing trend after 14 d of treatment, but significantly decreased after 21 d (*P* < 0.05).

The gene expression of *AMPK*α and *AMPK*β showed the same pattern in *S. vetulus* after quercetin treatment. The expression of these genes was significantly increased in the low concentration quercetin group (1 mg/L or 2.5 mg/L) after 7, 14, and 21 d of treatment (*P* < 0.05). In the high-concentration quercetin (10 mg/L) group, the expression levels of both *AMPK*α and *AMPK*β decreased compared with those in the control group (Figure 6C,D). Conversely, the expression of *AMPK*γ exhibited a declining trend in quercetin-treated groups compared to the control group, regardless of treatment time and dose (Figure 6E).

As shown in Table2, multi-factor analysis results showed the activity of amylase and the expression of *AMPK* were significantly affected by the quercetin concentrations and treatment time (*P* < 0.05). The activity of cellulase was significantly affected by the synergistic effect of concentration and time (*P* < 0.05).

**Table 2:** Summary of Two-Way ANOVA between Quercetin Concentrations and Treatment Time on the Enzyme Activity of Amylase and Cellulase and Expression of *AMPK*α, *AMPK*β, and *AMPK*γ in *S. vetulus*^a^

	parameter				
	amylase	cellulase	*AMPK*α	*AMPK*β	*AMPK*γ
C	<0.01	NS	NS	<0.05	<0.01
T	<0.01	NS	<0.01	<0.05	NS
C × T	NS	<0.01	NS	NS	NS

a C: quercetin concentrations; T: treatment time; C × T: quercetin concentrations × treatment time; and NS: no significant difference (*P* > 0.05).

## 4 Discussion

### 4.1 Quercetin Prolongs Lifespan without Negatively Affecting Growth and Reproduction in *S. vetulus*

In recent years, certain anti-aging drugs have been shown to extend the average and maximum lifespans of animals. For example, resveratrol significantly prolongs the average lifespan but has no significant effect on the maximum lifespan, of female silkworms.^25^ Moreover, α-lipoic acid increases the median lifespan of *C. elegans* with little effect on the maximum lifespan,^26^ while minocycline treatment strongly increases the median lifespan more than the maximum lifespan in female flies.^27^ In the present study, the maximum and average lifespans of *S. vetulus* were increased in the 1 mg/L quercetin group compared to that in the control group, with the maximum lifespan extended by about 22%. In addition, resveratrol is among the many flavonoids, including fisetin and catechin, that can extend the lifespan of animals to variable extents.^11,28,29^ Studies of resveratrol in aquatic animals have shown it to have anti-aging effects in the annual fish *Nothobranchius guentheri* but not in *Daphnia pulex* (*D. pulex*).^30,31^ This instability in the anti-aging effects of resveratrol was also found in a study of model organisms.^32^ Quercetin has been proven to prolong the lifespan of different species such as *C. elegans*, *Saccharomyces cerevisiae*, and *Drosophila melanogaster*.^8,9,18^ In the present study, quercetin was also found to prolong the lifespan of *S. vetulus*, meaning that the anti-aging effects of quercetin are probably more stable.

Dietary restriction is an acknowledged anti-aging method, and its regulatory mechanisms are conserved among multiple species.^33,34^ Previous studies of other anti-aging drugs such as vitamin E, caffeic acid, and rosmarinic acid have reported that these drugs can prolong the lifespan in several species.^35,36^ However, lifespan extension induced by dietary restriction and these drugs seems to come at the cost of reduced reproduction. In contrast, quercetin appears to benefit reproduction when the net reproduction rate is used to evaluate the number of offspring.^37^ In this study, there was the slight increase in the net reproduction rate in the quercetin-treated group, indicating that an appropriate dose of quercetin increased lifespan and had no negative effects on the reproduction of *S. vetulus*. This is a potential advantage of quercetin as an anti-aging drug. Intrinsic growth rate and generation time are good indicators of population permanence over time.^38,39^ Quercetin had no significant effect on intrinsic growth rate and generation time in all treatment groups, apart from those exposed to 10 mg/L quercetin, implying that the lifespan-extending concentrations of quercetin also had no negative effect on population growth in *S. vetulus*. The average lifespan, net reproduction rate, and intrinsic growth rate of *S. vetulus* were all significantly decreased in the 10 mg/L quercetin group compared with that in the control group, suggesting that, in small aquatic animals such as cladocerans, this concentration of quercetin may have adverse effects on individuals and populations.

### 4.2 Metabolic Pathways and Key Proteins Regulated by Quercetin in *S. vetulus*

In this study, we applied a proteomics approach to explore the anti-aging mechanism of quercetin at the molecular level in aquatic animals. There were a total of 156 DEPs identified, and these were subjected to GO and KEGG enrichment analyses. Through these enrichment analyses, it was found that the DEPs associated with quercetin treatment in *S. vetulus* corresponded mainly with changes in metabolism, including glycometabolism, energy metabolism, and sphingolipid metabolism.

Metabolism plays an important role in the regulation of aging,^40^ and previous studies have demonstrated that flavonoids can regulate glycometabolism in animals, thereby improving age-related diseases such as diabetes and non-alcoholic fatty liver disease.^41,42^ In experiments on cladocerans, it has been found that aging in *D. pulex* is closely related to glycometabolism.^43^ In the present study, the glycometabolism of *S. vetulus* was affected by quercetin. Two KEGG pathways involved in glycometabolism, starch and sucrose metabolism, and other glycan degradation pathways were significantly altered after quercetin treatment. These results showed that improvements in glycometabolism could prolong the lifespan of *S. vetulus*. Our results were similar to previous studies showing that there is a relationship between glycometabolism and longevity.^44−46^

Impairments in the ability to regulate energy metabolism occur during aging.^47^ Glycometabolism provides energy for basic life activities in an individual. The proteomic sequencing data showed that quercetin regulated the expression of enzymes related to sugar metabolism, such as 6-phosphogluconate dehydrogenase (6PGD), endoglucanase, β-mannosidase, β-hexosaminidase, α-amylase, endo-1,4-mannanase, cellobiohydrolase, and glucosylceramidase.^48^ In addition, the activities of amylase and cellulase increased after quercetin treatment. These results suggested that quercetin mediated the digestion, absorption, and metabolism of glycogen-based substances, improving the ability to regulate energy metabolism and providing more energy for the body.

The aging process is accompanied by changes in many key enzymes involved in glycometabolism. The 6PGD is associated with the pentose phosphate pathway and located downstream of the rate-limiting enzyme glucose-6-phosphate dehydrogenase (G6PD), which is involved in NADPH production and maintenance of cellular function.^49^ The overexpression of G6PD promotes cell growth and reduces oxidative stress,^50^ which are important factors in extending lifespan. 6PGD also assists G6PD in promoting cell growth and inhibiting cell death.^51^ Previous studies have shown that the activity of 6PGD decreased with increasing age in rat, which may be one of the important causes of aging in individuals.^52,53^ The results of this study showed that quercetin upregulated the expression of 6PGD and prolonged the lifespan of *S. vetulus*, suggesting that it may be the key enzyme associated with the quercetin-based regulation of glycometabolism, which delays aging.

In addition to glycometabolism, energy metabolism was also affected by quercetin. Both oxaloacetate and malate are essential members of the tricarboxylic acid (TCA) cycle. The TCA cycle plays an important role in energy metabolism, providing cells with a large amount of energy for life activities.^54,55^ Previous studies have observed reduced efficiency of the TCA cycle in aging-related neurodegenerative diseases.^56,57^ In the present study, the transmembrane transport of oxaloacetate and malate was significantly altered by treatment, implying that quercetin regulates the TCA cycle of *S. vetulus*.^58^ This is consistent with the results from other studies, which found that flavonoids benefit the TCA cycle.^59,60^

*AMPK* is a crucial cellular energy sensor in eukaryotes that maintains cellular energy homeostasis.^61^*AMPK* is activated when AMP/ATP is elevated, and it has one catalytic subunit (α) and two regulatory subunits (β and γ).^62,63^ Previous studies have shown that *AMPK* mediated quercetin-based induction of cancer cell apoptosis, alleviates mitochondrial dysfunction, and challenges the effects of obesity.^64−67^ Growing evidence suggests that *AMPK* plays an important role in longevity. Increased expression of aak-2/*AMPK*α in *C. elegans* and *Drosophila* extends their lifespans.^68,69^ Changes in *AMPK*γ have been found to have anti-aging effects on worms in studies of dietary restriction.^70^ This result was also found in our study. The expression of *AMPK*α and *AMPK*β was significantly increased, and the lifespan of *S. vetulus* was extended. This suggested that *AMPK* may also mediate the anti-aging effects of quercetin. This mediation of metabolic processes has also been found in studies of other anti-aging drugs, such as metformin, resveratrol, and aspirin.^71−73^

In the present study, sphingolipid metabolism also changed significantly after quercetin treatment. Sphingolipids are essential components of eukaryotic cell membranes, which maintain the structure stability, permeability, and fluidity of cell membranes^74^ and regulate cell growth, differentiation, aging, and apoptosis by acting as bioactive signaling molecules.^75^ Sphingolipids and sphingolipid metabolism enzymes have vast importance in aging and neurodegeneration.^76^ The modulation of sphingolipid metabolism has previously been revealed to be associated with the anti-aging effects of caloric restriction.^77^ Studies have shown that as sphingolipids, ceramides, and ceramide synthases regulated the lifespan of *Drosophila* and *C. elegans*.^78,79^ In our experiments, as one of the ceramide synthases, the expression of glucosylceramidase was significantly increased after quercetin treatment. These results indicated that quercetin improved sphingolipid metabolism by regulating sphingolipid metabolism enzymes. In addition, ceramides play an important structural role in caveolae and plasma membrane rafts,^80^ which are critical substances in the development and treatment of aging-related diseases.^81,82^ Caveolae and plasma membrane rafts also significantly changed in our quercetin groups.

In this study, quercetin inhibited the protein expression of Lamin A and activated protein expression of Klotho. Mutations in the nuclear structural protein Lamin A are the main cause of premature aging and participate in the progress of aging in healthy individuals.^83^ The *klotho* gene encodes a single-pass transmembrane protein and is considered to be an aging suppressor gene. Overexpression of Klotho protein in mice extends their lifespan.^84^ However, the role of Lamin A and Klotho in crustaceans remains unclear. The results of this study suggested that quercetin directly regulated the expression of anti-aging-related proteins.

## 5 Conclusions

Overall, we assessed the lifespan and growth parameters of *S. vetulus* and sequenced the proteome after quercetin treatment. The results showed that quercetin prolonged the average and maximum lifespans, increased reproduction, and had no negative effects on growth parameters such as intrinsic growth rate and generation time in *S. vetulus*. The proteomics results highlighted that quercetin improved glycometabolism (starch and sucrose metabolism, other glycan degradation), energy metabolism (TCA cycle and *AMPK*), and sphingolipid metabolism. Furthermore, quercetin was shown to regulate the expression of the anti-aging-related proteins Lamin A and Klotho directly. Our results provided basic data for understanding the molecular mechanism associated with the anti-aging effects of quercetin.

## Notes

The authors declare that all data supporting the conclusion of this study are available within the article.
